# Improving protein–ligand docking and screening accuracies by incorporating a scoring function correction term

**DOI:** 10.1093/bib/bbac051

**Published:** 2022-03-14

**Authors:** Liangzhen Zheng, Jintao Meng, Kai Jiang, Haidong Lan, Zechen Wang, Mingzhi Lin, Weifeng Li, Hongwei Guo, Yanjie Wei, Yuguang Mu

**Affiliations:** 1 Shenzhen Institute of Advanced Technology, Chinese Academy of Sciences, Shenzhen, Guangdong 518055, China; 2 Shanghai Zelixir Biotech Company Ltd., Shanghai 200030, China; 3 National Supercomputer Center in Shenzhen, Shenzhen, 518000, China; 4 Institute of Plant and Food Science, Department of Biology, School of Life Sciences, Southern University of Science and Technology (SUSTech), Shenzhen, Guangdong 518055, China; 5 Tencent AI Lab, Shenzhen, Guangdong 518000, China; 6 School of Physics, Shandong University, Jinan, Shandong 250101, China; 7 School of Biological Sciences, Nanyang Technological University, 60 Nanyang Drive 637551, Singapore

**Keywords:** scoring function, machine learning, molecular docking, reversal virtual screening, virtual screening

## Abstract

Scoring functions are important components in molecular docking for structure-based drug discovery. Traditional scoring functions, generally empirical- or force field-based, are robust and have proven to be useful for identifying hits and lead optimizations. Although multiple highly accurate deep learning- or machine learning-based scoring functions have been developed, their direct applications for docking and screening are limited. We describe a novel strategy to develop a reliable protein–ligand scoring function by augmenting the traditional scoring function Vina score using a correction term (OnionNet-SFCT). The correction term is developed based on an AdaBoost random forest model, utilizing multiple layers of contacts formed between protein residues and ligand atoms. In addition to the Vina score, the model considerably enhances the AutoDock Vina prediction abilities for docking and screening tasks based on different benchmarks (such as cross-docking dataset, CASF-2016, DUD-E and DUD-AD). Furthermore, our model could be combined with multiple docking applications to increase pose selection accuracies and screening abilities, indicating its wide usage for structure-based drug discoveries. Furthermore, in a reverse practice, the combined scoring strategy successfully identified multiple known receptors of a plant hormone. To summarize, the results show that the combination of data-driven model (OnionNet-SFCT) and empirical scoring function (Vina score) is a good scoring strategy that could be useful for structure-based drug discoveries and potentially target fishing in future.

## Introduction

Molecular docking is a useful tool for structure-based drug discoveries [1–4]. Scoring function is one of the most important components of a docking application [1]. A well-balanced scoring function should have the following three abilities. First, it should be fast and accurate to score and rank the large number of docking poses generated in docking simulations [1, 5, 6]. From the application aspect, accurate ligand pose selection would guide inhibitor design, agonist design, and enzyme–substrate catalytic mechanism exploration [7–10]. Second, a good scoring function should be able to screen the compound libraries, select the right binding poses and identify the active molecules, which are usually an extremely small portion of libraries having affordable computation resources and calculation times [5, 6, 11]. This ability would facilitate the ‘hits’ molecules identification via high throughput virtual screening at quite an early stage in a drug discovery project for small molecules. Finally, good scoring should allow strong binding between a small molecule and its native receptor but weak binding toward other off-target proteins [12]. Naturally, by adopting the reverse virtual screening pipeline, the capacity to identify the native receptor for a small molecule would be extremely useful in small molecule off-target evaluation, drug safety evaluation and target fishing [12–15]. The identification of native targets for natural products [15] or hormones (both in animals or plants) is an important area assuming there exists a reliable reverse screening pipeline that would help shortlist possible binding proteins for the query compound with a relatively lower false positive rate. Moreover, to locate the true target protein, limited wet lab experiments would be required. The current scoring functions generally are quite good at the first direction but not the last two. Some of the most successful traditional scoring functions for docking and screening tasks are Vina score (in AutoDock Vina [16]), ChemPLP (in GOLD [17]) and Glide score (in Glide [18]); however, they still have quite a high false positive rate during screening [6]. More recently developed machine learning (ML) and deep learning (DL) scoring functions were reported to have higher scoring ability for crystal protein–ligand complexes [19–28]. However, for docking and screening tasks, many of these functions are even not better than traditional scoring functions such as Vina score, Goldscore and ChemPLP [28, 29]. Traditional scoring functions, such as force field-based, statistical-based or empirical scoring functions-based, are mostly trained on datasets formed by experimentally determined high-quality protein–ligand complex structures, whereas docking poses are not observed [16, 30–32]. The limitation is that, when ranking the docking poses, the false positive poses (those are far away from the native pose) would be selected because the scoring functions cannot separate them from the native pose [5, 6]. Another limitation of the traditional scoring functions is that they are mostly expressed by over-simplified linear energy combinations; thus, they lack the ability to learn from corner cases and large-scale decoy datasets. A recent study [33] proposed a scoring function by building ML models to combine energy terms in ten traditional scoring functions for highly accurate screening tasks; however, the docking ability of the scoring function is not tested.

Both ML and DL models have the ability to capture high-level complexities in protein–ligand complexes and have the potential to formulate a powerful scoring function for more practical docking and screening tasks [20, 21, 29]. Previous models such as OnionNet [19], OnionNet2 [20], Pafnucy [34], Kdeep [35] and RosENet [23] have been reported to have high scoring and ranking scores; however, their performance on virtual screening is not as good as expected [28, 29], possibly because of the over-fitting to the experimental complex structures datasets. A recent docking application Gnina [22, 36] adopts a 3D convolutional neural network (CNN) model [37] to provide a score to the docking poses. It can largely improve both redocking and cross-docking task performance but its performance for screening power is not evaluated. The idea of training a DL model with docking poses in a suitable manner appears to be a good approach to obtain a more accurate scoring function [38]. Previous methods such as Δ_vina_XGB [39] and Δ_vina_RF20 [40] were trained on docking decoy datasets. Their learning labels were artificial binding affinities calculated as per the docking pose root mean square deviation (RMSD) and Vina scores with the hypothesis that these poses having larger RMSD should be labeled to have a lower binding affinity [40]. Inspired by these prior studies and our own DL exercises [19, 20], we suggest that the robustness of Vina score could be optimized by augmenting a ML-based score term. Accordingly, the screening power and power to differentiate the ‘good’ molecules from ‘bad’ ones could be enhanced. Here, in this study, a scoring model (OnionNet-SFCT) is built based on an AdaBoost random forests [41, 42] trained on docking poses with a broad range of deviations (RMSD) from native poses (the crystal ligand conformation). Unlike what the authors did for developing Δ_vina_XGB and Δ_vina_RF20, we did not apply artificial labels in model training; instead, only the RMSD values were used as labels [38], and the trained model works as a scoring function correction term (SFCT). The input features of this model are the multiple layers of protein–ligand intermolecular contacts (between protein residues and ligand atoms) [19, 20, 43]. The performance of adding OnionNet-SFCT term to the Vina score is verified by docking (in particular redocking and cross-docking) tasks, virtual screening tasks and reverse docking tasks.

Using OnionNet-SFCT, the top1 pose docking success rates of AutoDock Vina are largely improved. When the docking box center is predefined, the top1 pose success rate of AutoDock Vina increases from 70.5% to 76.8% for the redocking task using the PDBbind v2016 coreset [44] and from 32.3% to 42.9% for cross-docking task. Furthermore, OnionNet-SFCT+Vina increases the docking power by 3% for top1 poses with the CASF-2016 benchmark [6], and it greatly improves the screening power by almost doubling the enrichment factor of AutoDock Vina. Moreover, equipped with OnionNet-SFCT, the enrichment abilities of AutoDock Vina and Gnina on DUD-E [45] and DUD-AD [11] benchmarks are largely improved. Furthermore, we combine the OnionNet-SFCT correction term with other scoring functions in LeDock [46], iDock [47] and Gnina [36], as well as witness the increased redocking and cross-docking success rates, indicating that it could function as a general scoring term that is beneficial for different scoring functions. In a reverse docking task of abscisic acid (ABA) [48] on the *Arabidopsis thaliana* genome-wide proteins, OnionNet-SFCT+Vina score scheme detected 4 known targets (there are 14 known abscisic acid binding proteins) in the top-ranked 10 proteins. None of the known targets can be identified in the top-ranked 10 proteins only by the Vina score.

To summarize, the ML model that we developed can greatly improve the performance of docking applications in many tasks combined with traditional scoring functions. The OnionNet-SFCT model could be accessed via the github repository (https://www.github.com/zhenglz/OnionNet-SFCT.git).

## Method

### Datasets

We used protein–ligand complexes structures with experimentally determined binding affinity data (*K_i_*/*K_d_*) from PDBbind v2018 [44] (http://www.pdbbind-cn.org/download/pdbbind_2018_intro.pdf), as well as docking poses generated based on these protein–ligand complexes as training, validation and test sets for the ML scoring model development (Table 1). The dataset splitting scheme is similar to a previous study [19] by our group.

**Table 1:** The datasets included in this study

SN	Name	Source	# Samples	Notes
1	Train	PDBBind v2018 general set	12 906	Crystal structures
2	Validate	PDBBind v2018 refineset	1000	Crystal structures
3	test1	CASF2016 coreset	285	Crystal structures
4	test2	PDBBind v2013 coreset	195	Crystal structures
5	docking (train)	Docking poses based on train	10 208 × 90	Docking poses generated by iDock
6	docking (validate)	Docking poses based on validate	978 × 90	Docking poses generated by iDock
7	docking (test1)	Docking poses based on test1	285 × 90	Docking poses generated by iDock
8	docking (test2)	Docking poses test2	195 × 90	Docking poses generated by iDock
9	redocking (validate)	validate set	292	Crystal structures
10	redocking (v2016 coreset)	test1 dataset	285	Crystal structures
11	cross-docking	3D DISCO cross-docking benchmark [48]	4399	Cross-docking protein-ligand structures
12	CASF2016	CASF2016 docking and screening decoys [6]	57 targets	Docking and cross-docking poses
13	DUD-E	DUD-E actives and decoys [44]	102 targets	Docking poses generated by AutoDock Vina and Gnina
14	DUD-AD	DUD-AD actives and decoys [11]	102 targets	Docking poses generated by AutoDock Vina and Gnina

First, we selected the two core sets of the protein–ligand structures in CASF-2016 (test1 in Table 1, 285 samples) and CASF-2013 (test2 in Table 1, 195 samples) as test sets. These two test sets are manually calibrated high-precision datasets in which the properties of the binding pocket, the binding strength and the sizes of the ligands are relatively uniformly distributed; they have been used as standard test sets in multiple other studies [19, 21, 35]. Note that there are 107 overlapping samples in these two test sets.

PDBbind v2018 dataset has a total of 16 126 protein–ligand complexes; it can be divided into two independent data sets: the refined (4463) and general sets (11663). We excluded all samples with binding strength determination criteria of IC50 and short peptides as ligands. The primary concern is that the binding measurement strength by IC50 has a large difference with *K_i_* or *K_d_*, which is not suitable for mixing. Other method such as ΔVinaRF20 also excluded the IC50 data. To fairly compare with them, we did not include IC50 data here. Moreover, short peptides have more rotatable bonds, which are more difficult to predict and tend to increase noise. After excluding short peptides and IC50 samples, there are 13 279 samples left in the general and refined sets. Next, we removed all complexes in the two test sets (373 unique PDB complexes)from PDBbind v2018 refined set, resulting in 12 906 samples. Then, 1000 samples are randomly selected from the remaining samples in the refined set. These 1000 samples serve as the validation set samples, which was the same subset in OnionNet. After removing the test and validation sets, the remaining 11 906 samples serve as training samples (as used in OnionNet). Table 1 lists the number of samples in each set. For all samples in the test, training and validation sets, we removed the water molecules and metal ions from complexes before docking or featurization. The PDB entries of samples in Datasets 1–4 (in Table 1) are listed in the Supplementary Table.

For the abovementioned four datasets (train, validate, test1 and test2), docking simulations were used to generate docking poses. The docking poses (datasets 5–8 in Table 1), in addition to the native poses in the protein–ligand complexes crystal structures, are collected to train and validate our AdaBoost Random Forest-based scoring model OnionNet-SFCT. Here, we used iDock v2.2.1 to generate docking poses. iDock is a docking tool that adopts the same scoring function used in AutoDock Vina in the docking process and implements the RF score model for the output poses scoring. For each protein–ligand complex, the ligand was first extracted and prepared using AutoDock MGLTools 1.5.4 [7] (http://mgltools.scripps.edu) and prepare_ligand4.py to assign polar hydrogen atoms and convert the format from pdb to pdbqt. Then, the water molecules, metal ions, and any other non-ligand molecules were removed from the protein structure, which was then converted to pdbqt from the pdb format using AutoDock MGLTools prepare_receptor4.py script. The center of binding pocket (in Cartesian space) of the receptor (protein) was set as the geometry center of the native pose of ligand in the complex structure. Furthermore, the docking box size for all three dimensions was 15 Å, and other parameters were set as default values. For each complex, we performed ten independent repeats to generate a total of 90 poses (nine poses per run). All the docking poses generated by iDock here are used for OnionNet-SCT model training, validating and testing, but not for redocking and cross-docking or screening performance evaluation.

Note that 871 samples (849 in the training set and 22 in the validation set) failed to generate docking poses. Thus, we only obtained docking poses for remaining 10 208 and 978 protein–ligand complexes in the train and validation sets (Table 1), respectively. For each docking pose, RMSD with respect to its native pose was calculated using obrms in OpenBabel v2.2.3 [49]. Moreover, to evaluate the docking and screening powers, we adopted the popular CASF2016 docking decoys and screening decoys for scoring. The docking and screening powers are calculated based on Python script obtained from the CASF-2016 package [6].

### Redocking and cross-docking protocol

To evaluate the docking performance of our scoring model, we adopted three independent datasets for redocking scoring and cross-docking tasks. In redocking scoring tasks, the ligand in the crystal structure was extracted and redocked in the original ligand-binding pocket in the receptor; furthermore, deviations were calculated for each docking pose with respect to the native pose. In the redocking protocol, the docking success rate is defined as the ratio of samples where the RMSD of the top-ranking pose is <2Å [6]. Moreover, the average of RMSD values of the top-ranking poses for all protein–ligand complexes is computed. The smaller the average top-ranking pose RMSD is, the better the scoring function or scoring model performs. For redocking tasks, we adopted two datasets: redocking (validate) and redocking (v2016 coreset). In the first dataset, the complexes were randomly selected from the validation set; they were then used to confirm the redocking performance. Moreover, the second dataset contains 285 complexes used in CASF-2016.

As for cross-docking tasks, it is based on a standard benchmark (3D DISCO [50]), where 95 different target proteins with an average ~46.3 ligands per target extracted from the PDB entries (same protein but different PDB entries) to form an ‘artificial’ set with 4399 protein-ligand complexes. In each ‘artificial complex’, the receptor structure is a predefined structure (used in DUD-E dataset), called ‘representative receptor structure’. The ‘native pose’ of the ligand was generated as following: superimposing the protein structure (in the same PDB entry as the ligand) to the ‘representative receptor structure’ together with the ligand. The superimposed ligand was taken out and combined with the ‘representative receptor structure’ to form the so called ‘artificial complex’. We assume that the ligand pose in the ‘artificial complex’ is correct or ‘near-native’. Then, we cross-dock the ligand back into the ‘artificial complex’ pocket, and calculate the RMSD values of docking poses, and also use RMSD ≤ 2 Å to evaluate whether the docking pose is a ‘near-native’ pose or not. The detailed information of 3D DISCO could be reported in Wierbowski’s study [50].

We augmented the scoring model OnionNet-SFCT to the scoring functions in four different docking engines (AutoDock Vina, iDock, LeDock and Gnina). To evaluate the redocking performance of OnionNet-SFCT in combination with different docking applications, the docking poses are generated by that docking engine. For all docking experiments, the receptor was prepared by removing water molecules, ions, and metal ions as well as other small molecules. The ligand was then extracted from the original complex structure. For both Vina and iDock, all hydrogen atoms were removed, and then polar hydrogen atoms were added. Except the docking pocket definitions, we then followed the standard docking protocol for the four docking engines with their default scoring function and parameters.

For each protein-ligand crystal complex in different data sets, the ligand is extracted and docked into its corresponding protein pocket (the same PDB entry) only once using either iDock (nine poses), LeDock (less than 20 poses), Vina (20 poses) or Gnina (less than 20 poses) to generate docking poses. For the docking poses generated by different docking applications, they are rescored and ranked (by OnionNet-SFCT or OnionNet-SFCT with the docking scores generated by corresponding application) to evaluate the docking pose ranking ability of different scoring strategies.

Both redocking and cross-docking performances were separately evaluated for the original docking scoring functions, the scoring model OnionNet-SFCT, and the combination of them.

For Vina, two types of docking box are defined, one is centered at the geometry center of the original ligand with a 15 Å lenght cubic region, another is centered at the protein geometry center with a 100 Å length cubic region (Experiment 1 and 2 in Table 2). For iDock docking, we used the default parameters for sampling; however, the docking box was adjusted by extending the reference ligand region (in 3D space to 6Å) to mimic the ‘autobox’ setting (Experiment 3 in Table 2) in docking application such as Gnina. For LeDock, a docking box with 15 Å for all three dimensions are used (Experiment 4 in Table 2). In terms of Gnina (Experiment 5 in Table 2), the postscoring mode was used; furthermore, the default CNN scoring function for pose RMSD estimation (the CNN pose score) was used to ranking the poses.

**Table 2:** The docking pocket setting in different docking simulations

Experiments SN	Docking engine	Pocket center	Pocket size setting
1	AutoDock Vina	Original ligand center	15 Å for all three dimensions
2	AutoDock Vina	Receptor center	100Å for all three dimensions
3	iDock	Original ligand center	Autobox, with ligand extend 6Å
4	LeDock	Original ligand center	15Å for all three dimensions
5	Gnina	Original ligand center	Autobox, with ligand extend 6Å
6	Gnina	Original ligand center	15Å for all three dimensions

### DUD-E benchmark and DUD-AD benchmark

To evaluate the screening performance of OnionNet-SFCT for larger screening tasks, we adopted the DUD-E [45] (Accessed on 8 May 2021) and DUD-AD benchmarks [11] for docking and rescoring, respectively. For all 102 targets in the DUD-E benchmark, there were ~224 active molecules and ~11 200 decoy molecules per target. Although the DUD-AD benchmark was compiled to use the active molecules of the other 101 targets as decoys for a specific target, it is designed to remove the potential bias in the DUD-E dataset. For both datasets, for each target, we docked both active and decoy molecules to the receptor using the AutoDock Vina and Gnina and used OnionNet-SFCT (or combined scoring strategies) to rescore the docking results. The docking protocols are same as previous redocking or cross-docking experiments 1 and 6, as listed in Table 2. For virtual screening with Gnina, the CNN affinity score of the top-ranked pose by the CNN pose score was used to rank the ligand or decoy. For screening performance evaluation, for each ligand, the best score of the generated poses (not higher than 20) is used to represent the ranking score of the ligand. The screening performance of different scoring methods was then measured by the enrichment factor at different level as well as the area under curve (AUC) value.

### Reverse docking protocols

We downloaded the proteome-wide structure predictions (27 434 proteins) of *A. thaliana* from the AlphaFold protein structure database (https://alphafold.ebi.ac.uk/download) [51, 52]. The target small molecule is a plant hormone ABA [48], which has around 14 known binding (UniProt) targets recorded in the GO database [53] (https://www.ebi.ac.uk/QuickGO/annotations?goUsage=descendants&goUsageRelationships=is_a,part_of,occurs_in&goId = GO:0010427&taxonId = 3702&taxonUsage = descendants) (access date 15 August 2021). The ABA structure is obtained from the crystal structure A8S in PDB entry 3 K90 [48] and is prepared using MGLTools prepare_ligand4.py script to add polar hydrogens. The proteins from AlphaFold2 predictions are then prepared using MGLTools prepare_receptor4.py script to add polar hydrogens. The binding sites (or binding site residues) are then determined using PointSite [ 54]. For each protein structure, PointSite assigns a probability score (between 0 and 1.0) for each atom. When the score is >0.5, we tend to believe this atom is located around with a binding pocket. Based on the per-atom pocket probability scores, we calculated the residue level scores by averaging the scores of atoms in a residue and ranked the residues by their scores. Then, a list of nonshort-range pocketable residues (where the residue-level score is >0.5) is generated in the descending order by their per-residue scores. If two residues are separated by >4 residues (such as residue *i* and residue *i* + 5) in the sequence, they are nonshort-range residues. We select a maximum of top-ranked 10 residues from the list. For each residue, we defined the geometry center of the residue as the pocket center, and then a box size as 15 Å for all three dimensions. There are in total 16 378 proteins with at least one pocket site residue. AutoDock Vina was used to dock the molecule ABA in the protein pockets centered on a maximum of 10 binding site residues; furthermore, the binding strength between the protein and ABA is defined by the lowest energy (Vina score or OnionNet-SFCT+Vina) of all docking poses. The docking parameters used in the reverse docking are obtained from Table 2 and Experiment 1. Therefore, the ABA-binding proteins can be predicted by ranking the 16 378 proteins based on their binding strengths to obtain the tightest binders.

### The RMSD prediction model

We collected both the crystal complexes and docking poses generated by iDock for OnionNet-SFCT model training (Datasets 1–8 in Table 1). The input features of a complex structure were calculated based on OnionNet2 with the modified number of shells and distance gap between shells (14 shells and distance gap of 1.5 Å). The labels of these complexes are the RMSD values of ligand pose with respect to their native pose. Therefore, for the crystal complexes, the RMSD is 0Å. While training, we excluded all complexes whose RMSD value is >10Å because large RMSD poses are generally quite far away from the binding site and may introduce noises to the scoring model. Then, we trained an AdaBoostRegressor [41] model with random forest [42] as the base estimator using the scikit-learn ensemble module. AdaBoost is known as a meta-estimator that first creates a regressor on the training data, and then adds additional estimators and weights to focus more on the difficult samples for the subsequent estimators. It has been confirmed to be a powerful model for many tasks [55–57]. Here, in this study, the AdaBoostRegressor is based on 10 base estimators. Each base estimator is a random forest model formed by 50 trees with max_features = 512, max_depth = 50 and oob_score = True (Supplementary Table S1). Moreover, we attempted CNN models for the RMSD prediction, although the root mean squared error (RMSE) of the test sets (with their docking pose complexes) was relatively low (Supplementary Table S1). Their performance for the redocking tasks on redocking (validate) dataset was not better than the AdaBoost model with the random forest model, indicating that the CNN model tends to overfit the data in our case.

Finally, as shown in Equation 1, we applied a simple linear combination of the correction term ($S(\mathbb{R}),$ from our OnionNet-SFCT model) and the original scoring function ($P(\mathbb{R})$) (could be a scoring function from Gnina, LeDock, Vina or iDock),

(1) \begin{equation*} E\left(\mathbb{R}\right)=\alpha \ast P\left(\mathbb{R}\right)+\beta \ast S\left(\mathbb{R}\right) \end{equation*}

where $\mathbb{R}$ is the coordinates of the complex structure formed by the protein and ligand poses. Although the unit of the correction term $S(\mathbb{R})$ (predicted RMSD, in Å) is different from that of the docking scores, generally the free energies (kcal/mol). On the other hand, these two terms have some common features. When $S(\mathbb{R})=0$, it takes only one possible state (the native state), and the larger the $S(\mathbb{R})$ values, the structures deviate further away from the native state and adopt more diverse states. The same goes for free energies of protein-ligand binding states. Therefore, the RMSD value could be viewed as a ‘pseudo-free energy’ term and thus it is meaningful to combine two terms together.

A similar combining strategy was applied in another study (ΔVinaXGB) [39], where they used the binding affinity minus (RMSD−1) × 0.5 as the train label for part of the samples. The weight parameter α and β was optimized using the OnionNet-SFCT+Vina scoring strategy on the redocking (validate) set, and *α,β* = 0.5 was reported to be a good selection for redocking (Figure S1 in Support Information) for Vina score; thus, we used *α,β* = 0.5 for all the analysis unless stated otherwise. The weighting factors may not necessarily be optimal for other scoring functions (such as those in LeDock and Gnina), although their scoring values may locate in the similar range. For simplicity, we only optimize the weighting factors for Vina score, but not LeDock or Gnina, to indicate that the idea itself works even though no delicate fine-tuning is performed. However, future works may be required to fine tune the weighting factors for all other scoring functions to obtain more accurate scoring strategies.

## Results and discussion

### OnionNet-SFCT improves redocking and cross-docking performance

At the first place, a good scoring function should be able to identify the native or near-native poses during docking simulations [6]. The performance of Autodock Vina with or without rescoring using OnionNet-SFCT+Vina on redocking and cross-docking tasks is reported when then ligand-binding pocket is defined or unknown [36].

**Figure 1:**
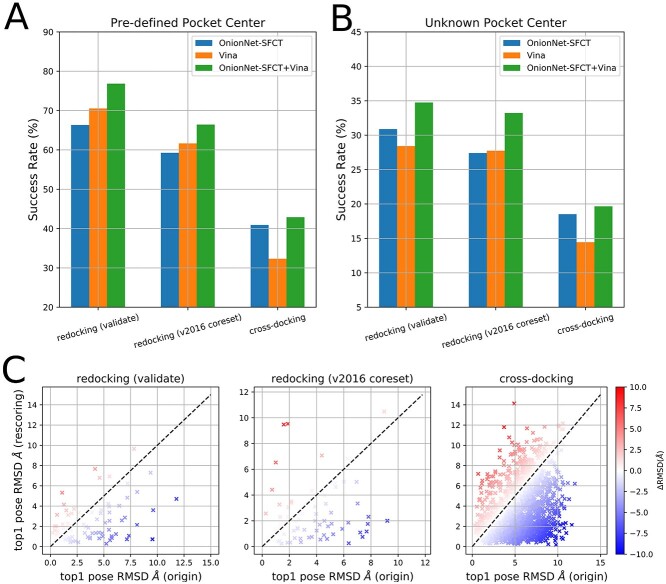
The performance of different scoring models (OnionNet-SFCT only, Vina score only and OnionNet-SFCT+Vina) on redocking and cross-docking tasks. (A–B) the success rate of different scoring methods with ligand binding pocket was predefined (A) or unknown (setting receptor geometry center as the docking box center) (B). (C) The RMSD values of the top-ranking poses for each protein-ligand complex with (*y*-axis) or without (*x*-axis) OnionNet-SFCT term.

For AutoDock Vina, the success rates of top1 poses are 61.6% and 70.5% for these two redocking datasets (validate and v2016coreset), when the exhaustiveness value is 64 and the docking box sizes is set as 15Å for all three dimensions in docking. If docking poses were rescored using OnionNet-SFCT energy term alone, the success rates for top1 poses were not improved in the redocking datasets; even worse, it is less accurate than the Vina score. As for the cross-docking task, OnionNet-SFCT shows higher success rate compared to the Vina score. However, if rescored by a combined OnionNet-SFCT+Vina function, the top1 pose success rates could be improved to 66.4% and 76.8%. In terms of the cross-docking task, the top1 pose success rate increases from 32.3% to 42.9% after rescoring by OnionNet-SFCT+Vina. iDock then implements the same scoring function as AutoDock Vina but with a redesigned acceleration scheme to speed up docking simulations. Similarly, OnionNet-SFCT alone as the rescoring energy is not capable to improve the redocking performance. If combining Vina score with the correction term, we, however, observe the performance enhancement in the rescoring (top1 pose success rate from 63.0% to 70.2% and 71.2% to 78.6% for redocking (validate and v2016coreset) and cross-docking (from 35.8% to 44.9%) tasks (Figure S2A).) For all the three datasets, the RMSD values for the top1 poses (generated either by AutoDock Vina or iDock) largely decreased after rescoring by OnionNet-SFCT+Vina (Figure 1C and Figure S2 in Support Information), indicating that OnionNet-SFCT+Vina tends to select lower RMSD poses than Vina and iDock. When the ligand-binding site is not defined, it is a more tough task for docking, although the binding site could be predicted using physics-based or deep learning-based algorithms. However, in this study, we set the geometry center of the receptor (the protein structure) as the docking box center and use a very large box size of 100 Å to mimic the setting in the Gnina evaluation experiments [36]. Under such circumstances, additional sampling efforts should be placed, and the docking success rate drops [36]. For AutoDock Vina, the top1 pose success rates of the two redocking datasets are 28.4% and 27.7%, whereas the success rate of the cross-docking task is only 14.4%. By adding the correction term OnionNet-SFCT, the success rates increase to 34.7%, 33.2% and 19.6% (Figure 1B). Comparing the experiments with the pre-defined binding pocket center, the improvement is less obvious, partially because OnionNet-SFCT is trained with poses RMSD values of <10 Å; however, in blind docking conditions, many docking poses are quite far away from the native poses with considerably larger RMSD values.

In summary, by adding OnionNet-SFCT as a scoring term, the new score (OnionNet-SFCT+Vina) is more accurate for selecting the lower RMSD poses from docking poses.

### OnionNet-SFCT improves redocking and cross-docking performance for different docking applications

Currently, most scoring functions for molecular docking are trained on crystal structures [16, 19, 23, 30, 34, 35]. These scoring functions thus primarily obtain the ‘true’ information from experimental protein–ligand complexes. However, during virtual screening exercises, large number of poses (or decoys) are generated; they are not previously seen in the scoring function training steps. It is hypothesized that the accuracy of scoring functions that are trained with decoys could be improved [36, 39, 40, 50]. Gnina, e.g. a forked version of AutoDock Vina and Smina [58], predicts the binding affinities and pose RMSD values in the same framework; moreover, it outperforms its ancestor Vina. CNN-based scoring function used in Gnina was trained using a large amount of docking/decoy poses in the Crossdock2020 benchmark [50]. In a same spirit, the augmentation of OnionNet-SFCT term to the Autodock Vina scoring function largely increases the overall scoring power. It is interesting to verify the generality of such chimeric scoring strategy. For this purpose, we attempted to add the correction term to the original scoring function of other docking applications (such as iDock, LeDock and Gnina), as shown in Equation 1.

**Figure 2:**
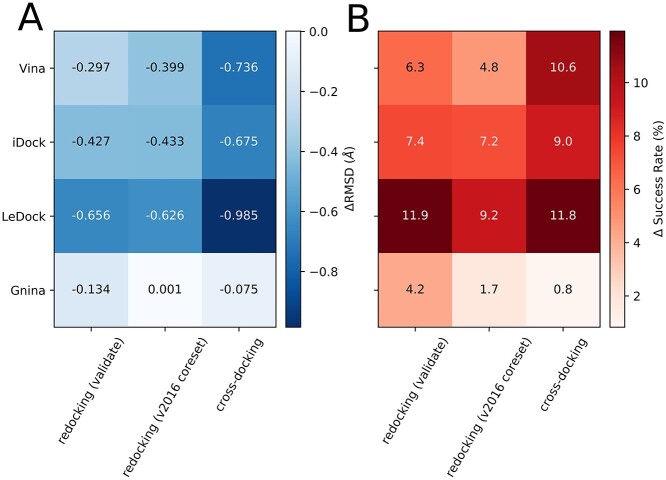
The performance of OnionNet-SFCT along with different scoring functions on redocking and cross-docking tasks. (A) The change of the average top-ranking pose RMSD values after rescoring with OnionNet-SFCT combined scoring functions. (B) The change of the success rate of the top-ranking poses after rescoring with OnionNet-SFCT combined scoring functions.

Clearly, for all docking applications, the chimeric scoring functions, by adding the correction term, improve the docking success rates for the top1 poses and the average top1 pose RMSD values (Figure 2). In particular, OnionNet-SFCT+iDock decreases the top1 pose average RMSD by 0.46, 0.443 and 0.6745 for redocking datasets and the crossdocking dataset; moreover, it largely increases the top1 pose docking success rates. Furthermore, LeDock is less able to rank the near-native poses with low energies; however, with the correction term, the docking success rate of OnionNet-SFCT+LeDock is largely improved on all three datasets. Furthermore, the top1 pose average RMSD values are decreased, particularly for the cross-docking dataset. Finally, the combination (OnionNet-SFCT+Gnina) only slightly decreases the average top1 pose RMSD and improves the top1 pose success rate for two datasets (PDBbind v2016 coreset and cross-docking dataset, Table 1). To summarize, the results suggest that OnionNet-SFCT has the potential to combine with other scoring functions for redocking and cross-docking tasks and for low RMSD pose selections.

### Improved docking power for Vina with OnionNet-SFCT on CASF-2016 benchmark

CASF-2016 is a popular benchmark for evaluating scoring functions [6]. It proposes four metrics (scoring power, ranking power, docking power and screening power) to assess the ability of scoring functions for pose ranking, selection and screening. The docking power represents the ability of a scoring function to select the near-native poses (RMSD of <2 Å with respect to the native pose) and rank them ahead as per the energies or predicted affinities [6, 39, 40]. In particular, a good scoring function would select the near-native poses in the top-ranking poses with a higher success rate (number of successful picked cases out of 285 protein–ligand complexes).

**Figure 3:**
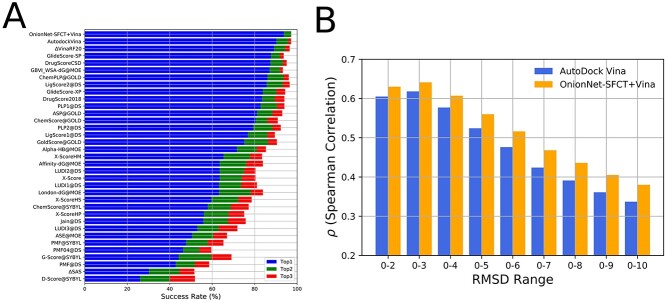
The docking power (A) and RMSD-energy correlations (B) of OnionNet-SFCT+Vina scoring strategy.

Based on the analysis of predictions provided by a list of scoring functions with the CASF-2016 benchmark, AutoDock Vina achieves the best docking power with a 90.2% success rate for the top1 poses among the traditional scoring functions, indicating that for 257 out of 285 protein–ligand complexes, the top1 poses are the near-native or native poses. Δ_vina_RF20 and Δ_vina_XGB were reported to achieve high success rate for 90% and 92%. Here, we calculated the docking power of the calibrated scoring function OnionNet-SFCT+Vina, which can obtain the highest success rate (93.7%) for the top1 poses, successfully identified the near-native poses for 267 protein–ligand complexes. Furthermore, another indication of good docking power is correlations between the energies or affinities with the pose RMSD values [6]. The new scoring function has higher correlations with RMSD values of poses compared to the original Vina score for all RMSD ranges (Figure 3D). To summarize, these results suggest that OnionNet-SFCT+Vina is a better scoring function for near-native pose selection.

**Figure 4:**
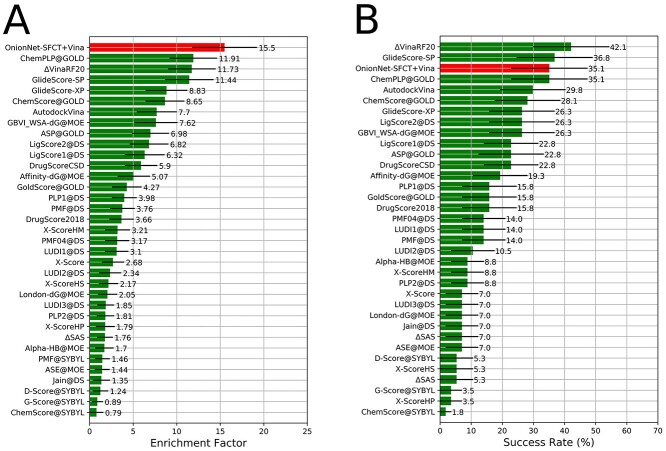
The screening power of the OnionNet-SFCT+Vina scoring strategy. (A) The enrichment factor (1%) of different scoring functions. (B) The success rate for identifying the best ligand in the top-ranking poses for different scoring functions. The confident intervals at 95% are indicated by black lines.

### Improved screening power for Vina with OnionNet-SFCT

In addition to the docking power, the screening power is another evaluation metric for scoring function assessment, which is adopted in scoring function development practices [5, 6]. The screening power shows the ability of a scoring function to enrich the active molecules at different levels and identify the best active molecules.

The enrichment factor, which is the concentration of the active ligands among the top-scoring docking hits compared to their concentration throughout the entire database [6, 11, 31, 45], is usually used for assessing the screening power of docking tools. In the CASF-2016 benchmark, OnionNet-SFCT+Vina achieves the highest average enrichment factor of 15.5 at 1% level, which is two times of Vina score itself (7.7) (Figure 4A). Furthermore, the average enrichment factor is much higher than other well-behaved scoring functions such as ChemPLP [17] (11.91) implemented in GOLD and Δ_vina_RF20 (11.73). What’s more, OnionNet-SFCT+Vina ranks after GlideScore-SP [18], similar to ChemPLP in identifying the best active molecules for the top 1% poses ((Figure 4B). The best-performed scoring functions are Δ_vina_XGB, Δ_vina_RF20 and GlideScore-SP in this assessment. In summary, OnionNet-SFCT+Vina is one of the top-ranking scoring strategies for screening tasks tested in CASF 2016.

### Improved screening performance with the DUD-E and DUD-AD benchmark

DUD-E benchmark is designed to fairly compare different scoring functions for screening. Certain scoring functions [31] are trained on part of it and tested with the remaining targets, and this type of evaluation would promote the scoring functions remember the ‘active’ molecules, thus including potential hidden bias [11]. Therefore, the active as decoy benchmark (DUD-AD) is designed to use the active molecules in other targets as decoys for a specific target. This DUD-AD benchmark is a more difficult screening dataset for scoring function evaluation. In this study, we do not train our OnionNet-SFCT neither with a part of the DUD-E dataset, nor the DUD-AD dataset to avoid potential bias.

**Figure 5:**
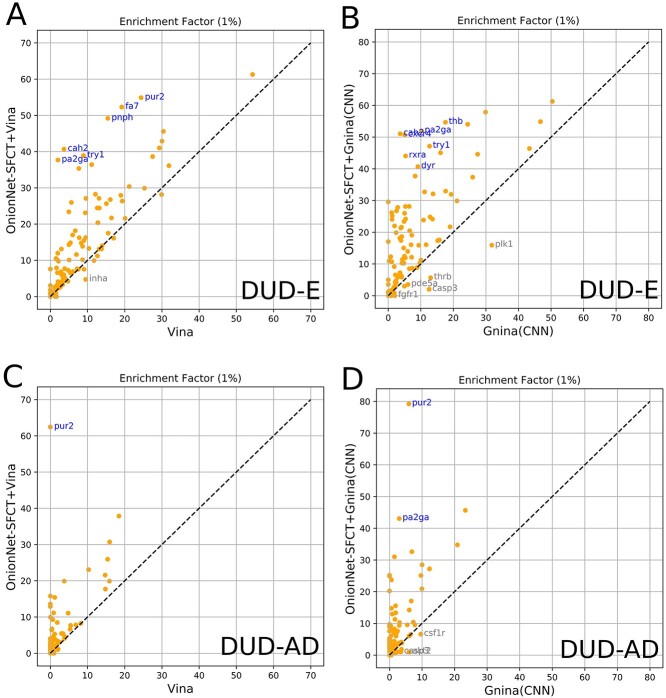
The per-target enrichment factor comparison with or without rescoring by OnionNet-SFCT+Vina or OnionNet-SFCT+Gnina on the two benchmarks DUD-E (A and B) and DUD-AD (C and D). For targets whose enrichment factors are largely increased (up to 30) with rescoring, the target names are labelled.

Here, we first report the screening performance of AutoDock Vina and OnionNet-SFCT+Vina on the DUD-E benchmark. The average enrichment factors (1%) for AutoDock Vina and OnionNet-SFCT+Vina is 8.823 and 15.544, respectively. For most targets, after rescoring (with OnionNet-SFCT+Vina), the enrichment factors are doubled or near doubled (Figure 5A). Similarly, OnionNet-SFCT improves the screening performance for Gnina (CNN scoring), the average enrichment factor (1%) is 18.687 and 7.932 with or without rescoring by OnionNet-SFT + Gnina, and the per-target enrichment factors increase by a large margin (Figure 5B). Here, we attempt to employ the similar docking pocket definitions for AutoDock Vina and Gnina for a fair comparison between the two docking applications. We notice that the average enrichment factor of Gnina does not greatly outperform AutoDock Vina, indicating that a deep learning scoring function good at docking (Gnina is originally designed for docking) is not necessarily good at screening. However, by rescoring with the hybrid scoring function equipped with OnionNet-SFCT, the screening performance of both AutoDock Vina and Gnina could be greatly improved.

We observe large improvements with rescoring on the unbiased DUD-AD benchmark. For docking results generated using AutoDock Vina, the average enrichment factor (1%) increased from 1.903 (Vina) to 5.223 after rescoring using OnionNet-SFCT+Vina (Table 3). Although for the docking poses from Gnina, with or without rescoring by OnionNet-SFCT+Gnina, the average enrichment factor (1%) is 8.502 and 2.332, respectively (Table 3). Note that for the DUD-AD benchmark, OnionNet-SFCT alone generally is more capable to have higher enrichment factors or ROC–AUC values.

**Table 3:** The screening performance of Vina, Gnina as well as OnionNet-SFCT model

		Enrichment factor (1%)	ROC-AUC				
	docking	Origin^*^	Rescore1^**^	Rescore2^#^	Origin^*^	Rescore1^**^	Rescore2^#^
DUD-AD	Vina	1.9029	5.2231	11.0166	0.4727	0.5491	0.6840
DUD-AD	Gnina	2.3322	8.5021	9.9505	0.5394	0.6415	0.6781
DUD-E	Vina	8.8246	15.5443	12.4287	0.6966	0.7242	0.6548
DUD-E	Gnina	7.9315	18.6873	13.9259	0.6799	0.7449	0.6765

^*^Original: the docking poses are selected and ranked by the original scores provided by the docking application (Vina or Gnina).

^**^Rescore1: the docking poses are selected and ranked by the hybrid scoring strategy OnionNet-SFCT+Vina or OnionNet-SFCT+Gnina.

^#^Rescore2: the docking poses are selected and ranked by the OnionNet-SFCT score function only.

**Figure 6:**
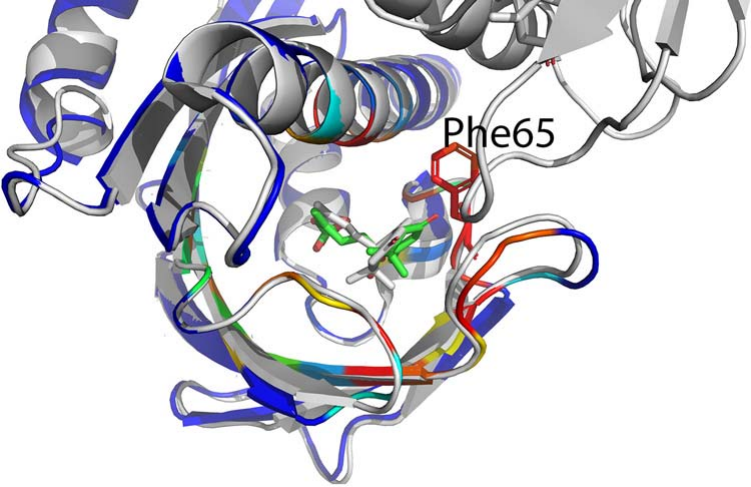
Comparison of binding modes in crystal structure (gray) and predicted by OnionNet-SFCT (green). The native protein structure is gray and binding site residue Phe65 is indicated by red sticks. The predicted protein structure is colored by the atom-level pocket probability scores (ranging from 0.0 to 1.0) where blue indicates low probability score and red indicates high probability score.

For each subset in the DUD-AD (or DUD-E) benchmark, the average enrichments and AUC scores largely increased after rescoring for all targets and targets in different subsets (Supplementary Table S1). The results therefore indicate that the OnionNet-SFCT+Vina scoring function or OnionNet-SFCT+Gnina scoring function are more accurate for screening tasks.

Comparing the performance increase in redocking and cross-docking tasks, OnionNet-SFCT+Vina or (OnionNet-SFCT+Gnina) achieves much higher accuracy. So why less significant docking power performance gain would lead to large screening power increase? We believe most docking programs in use are all quite good in scoring power and docking power, e.g. be able to find the native-like poses. And there is no big room for improving the docking power further. On the other hand, there is a large space for improving screening power, by separating decoy molecules from the true binders. In virtual screening, the binding affinity only, may not be a good target function, because some true binders have low binding affinity as well. In a protein–ligand docking task, when the majority poses are decoys (bad poses), the binding affinity score (Vina score in this case) would be more likely to make mistakes, resulting in high false positive rate. Thus, the ability to correct the Vina scores by putting penalty on the bad pose (RMSD prediction) would filter out the ‘bad’ poses and reduce false positive rate.

### OnionNet-SFCT could be applied for reverse screening

Reverse virtual screening is both useful in drug repositioning and drug rescue [12–14] and promising in identify potential drug targets [12–15]. With the open source of AlphaFold2 [51], the highly accurate predicted structures (with an average accuracy lDDT [59] around 85.0 in CASP14) open the gateway to the high-throughput structure-based drug discoveries and other functional analysis. In this study, we consider the reverse screening task against the stress responses related plant hormone ABA as an example to demonstrate the usability of OnionNet-SFCT. The *Arabidopsis thalian* protein structures were obtained from AlphaFold2 protein structure database [52]. The binding pockets were predicted using PointSite and docking poses of ABA generated by AutoDock Vina. Moreover, the predicted binding strengths were predicted by either using Vina score or OnionNet-SFCT+Vina.

Interestingly, using OnionNet-SFCT+Vina as the scoring function, out of the 14 known ABA receptors in *Arabidopsis thalian* proteome, we detected four targets in the top-ranked 10 proteins and eight targets in the top-ranked 100 proteins (Support Information). As for the first identified ABA target PYL9 (UniProt ID Q84MC7), the binding pose selected by OnionNet-SFCT+Vina is compared with the native pose solved in crystal structure (PDB entry 3OQU [48]) with RMSD = 1.9 Å (Figure 6). In comparison, among the 10 or 100 top-ranked proteins scoring by Vina score, none or 1 (PYL6) of the known ABA targets were identified, indicating that Vina score has high false positive rate for ABA target identification. The full list of the top-ranked 100 proteins as well as their binding scores calculated by Vina score or OnionNet-SFCT+Vina could be found in Supplementary Table.

To summarize, our scoring strategy significantly outperforms Vina score itself in the ABA reverse screening task. Although this is a demo example, it provides not only the ranking order but also the binding pattern predictions, indicating the potential usage of the OnionNet-SFCT+Vina score scheme for potential accurate target fishing application.

### Comparison with other models

The idea of modifying classical scoring functions by machine learning algorithms has been welcomed by other researchers. The $\Delta$_Vina_RF20 [40] model, which employed 20 descriptors, in addition to the AutoDock Vina scoring functions trained with random forest greatly improved scoring, ranking, docking and screening powers. Such strategy can be considered as machine learning-optimized empirical scoring functions. Recently, the energy components from classical scoring functions (such as Glide, GOLD and MOE) have been extracted and used as inputs to build new machine learning-optimized scoring functions and demonstrated large improvement in the screening power [33]. However, in our approach, rather than modifying the existing scoring functions terms, a new different score correction term, OnionNet-SFCT, is added. Different from our previous models (OnionNet and OnionNet2), which only fitted the binding affinities of crystal structures, the OnionNet-SFCT model is based on residue-atom contacts similar to OnionNet2 and it is trained on the artificial generated docking poses datasets (as did in $\Delta$_Vina_XGB [39]) and learns the docking pose qualities (RMSD values), it could be directly combined with various docking applications as a rescoring tool. Thus, the OnionNet-SFCT+Vina scoring strategy is designed to improve docking and screening powers. However, we also calculate the scoring and ranking power based on CASF-2016 benchmark [6]. The scoring power (Pearson R 0.428) and ranking power (Spearman R 0.393) of OnionNet-SFCT+Vina is significantly lower than those of $\Delta$_Vina_RF20 (0.816 and 0.75, respectively; Supplementary Table), indicating that a scoring function good at docking and screening may not necessarily also be good at native pose scoring and ranking. The same goes for Gnina [36], whose docking power is quite high, but screening power is relatively lower than Vina score. After detailed examination of the OnionNet-SFCT+Vina scoring and ranking results, we notice that the predicted RMSD values of many crystal poses are quite large, resulting in quite low scoring and ranking power. The main reason could be the pair-wise features used in this study may not fully capture the complete interactions between proteins and ligands. More well-balanced models for both scoring, ranking, docking and screening powers are still in need.

To check the overfitting and to validate the generalization capability of our model we have tested additional models trained on different nonoverlapping training sets with various similarity cutoff to the testing set (CASF-2016 core set) following Su’s work [60]. The outcomes have been added to the Support Information. We do observe some training set effects, e.g. the accuracy of the model on the test sets decreases with more dissimilar training sets (Table S2); similar as the docking power (Table S4). The scoring and ranking power (Table S3), however, increase with more diverse training sets. The reported screening power of our final model, in term of the enrichment factor, is found having no inflation effects with the training set.

**Figure 7:**
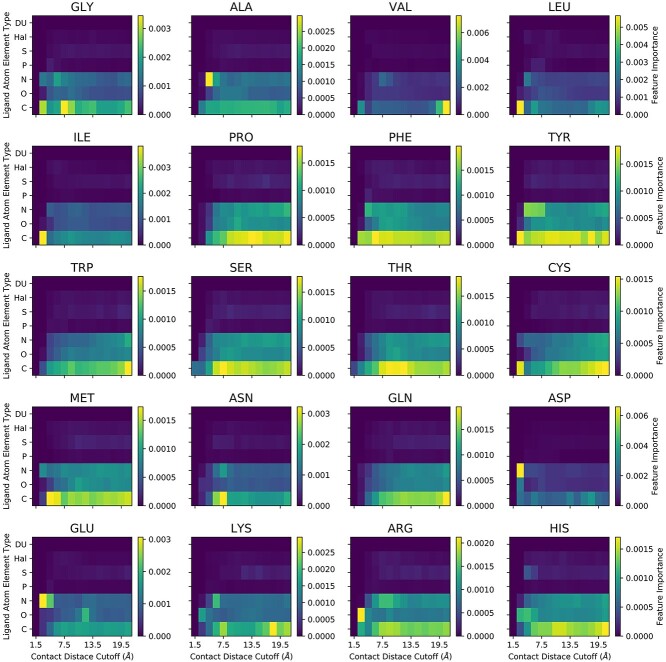
The feature importance of the OnionNet-SFCT model. The *x*-axis represents the contact distance cutoff, whereas the *y*-axis represents the element types of the ligand molecules. Each panel displays the importance of the interactions between different ligand atoms with a specific residue type. The color scales indicate the feature importance, with yellow color suggests the highest importance whereas the blue color indicates the lowest importance.

Moreover, the OnionNet-SFCT model is an intrinsic data-driven scoring function, learnt from known and artificial protein–ligand residue–atom pairs distance distributions [19, 43]. From the feature importance analysis (Figure 7), clearly OnionNet-SFCT could capture some short-range and-long range interactions between ligand atoms and protein residues. For example, the close contacts (around 3.0–4.5 Å) between nitrogen atoms in ligand molecules and the two acidic residues (aspartic acid and glutamic acid) tend to the most important interactions. Similarly, the contacts between oxygen atoms in the ligand molecules and the positively charged residues (lysine and arginine) are also identified as the most importance interaction patterns. These results suggest that the OnionNet-SFCT model could capture polar contacts or hydrogen bonds between the ligand and the receptor, and it learns the physical interaction rules itself but provides further complexity. Overall, it is speculated the combination of classical empirical scoring functions with machine learning-optimized knowledge-based scoring functions may have complementary nature and cover the additional aspects of the complicated protein–ligand binding processes *in vivo*.

## Conclusion

Scoring function is an important component in molecular docking. We designed a machine learning-based scoring function correction term for enhancing the prediction accuracies of traditional scoring functions such as Vina score. The OnionNet-SFCT model was trained using large-scale docking decoys generated by docking simulations based on experimental determined protein–ligand complexes; it borrowed the residue-atom multiple layer contacts from our previous work and adopted the AdaBoost random forests model to estimate the docking decoy RMSD values (with respect to their native poses). When working with Vina score for rescoring, the model is confirmed to be more accurate in redocking and cross-docking tasks; moreover, it largely improves the docking power and screening power on the CASF2016 benchmark. Next, on larger benchmarks (DUD-E and DUD-AD), the OnionNet-SFCT model is extremely accurate for screening. Furthermore, we incorporate the model into other docking applications (iDock, LeDock and Gnina) and find it can increase the redocking and cross-docking abilities for iDokc and LeDock to a large amount, as well as slightly increase the performance of Gnina, which already implemented a CNN-based scoring function trained on large-scale docking poses datasets. Lastly, using a reverse docking task, we prove the accuracy of OnionNet-SFCT+Vina score strategy by successfully identifying ligand’s natural receptors. To summarize, the OnionNet-SFCT model is a useful tool for structure-based drug discoveries.

**Key Points**

- A machine-learning model (OnionNet-SFCT) is come up to correct the scoring by physical or empirical scoring function (Vina score).
- The model shows good performance on docking related tasks (redocking and cross-docking).
- The screening accuracies are almost doubled when Vina score is equipped with OnionNet-SFCT.
- The combination of Vina score and OnionNet-SFCT could be applied for reverse screening.
- OnionNet-SFCT captures certain short-range polar interactions between the protein and the ligand.

## Supplementary Material

supplementary_table_R2_bbac051

## Author Biographies

**Liangzhen Zheng**, is a postdoctoral research fellow at Shenzhen Institute of Advanced Technology, Chinese Academy of Sciences. He is also a research scientist at Shanghai Zelixir Biotech Company Ltd. He is working on protein modeling and protein-ligand interaction modeling.

**Jintao Meng** is an associate researcher at Shenzhen Institutes of Advanced Technology, Chinese Academy of Sciences and National Supercomputing Center in Shenzhen. His research focus on high performance computing, deep learning and bioinformatics.

**Kai Jiang** is a research associate professor at Department of Biology, Southern University of Science and Technology (SUSTech). He is working on plant hormone signal transduction and development of plant growth regulators with chemical genetics.

**Haidong Lan** is a research scientist at Tencent AI Lab. He is working on high performance computing.

**Zechen Wang** is a master student at School of Physics, Shandong University. He is working on protein-ligand interaction prediction and drug molecule screening.

**Mingzhi Lin** is a senior engineer at Shanghai Zelixir Biotech Company Ltd. He works on high performance computing and infastructure design.

**Weifeng Li** is a full professor at School of Physics, Shandong University. He is interested in multi-scale simulations of biological systems and drug development.

**Hongwei Guo** is a chair professor at Department of Biology and the director of the Institute of Plant and Food Science at Southern University of Science and Technology (SUSTech). He is expertised in plant hormone signaling and small RNA biology.

**Yanjie Wei** is the executive director of Center for High Performance Computing at Shenzhen Institutes of Advanced Technology, Chinese Academy of Sciences. His research area is bioinformatics and high performance computing.

**Yuguang Mu** is an associate professor at School of Biological Sciences, Nanyang Technological University, Singapore. He is working on protein dynamics, molecular docking and drug discovery.
